# Viral Etiologies of Acute Dehydrating Gastroenteritis in Pakistani Children: Confounding Role of Parechoviruses

**DOI:** 10.3390/v7010378

**Published:** 2015-01-20

**Authors:** Muhammad Masroor Alam, Adnan Khurshid, Shahzad Shaukat, Muhammad Suleman Rana, Salmaan Sharif, Mehar Angez, Nadia Nisar, Uzma Bashir Aamir, Muhammad Naeem, Syed Sohail Zahoor Zaidi

**Affiliations:** 1 Department of Biotechnology, Quaid-i-Azam University, Islamabad 45320, Pakistan; E-Mails: ursmasroor@yahoo.com (M.M.A.); mnaeemqau@gmail.com (M.N.); 2 Department of Virology, National Institute of Health, Chak Shahzad, Park Road, Islamabad 44000, Pakistan; E-Mails: kadnan12@gmail.com (A.K.); vibgyors@yahoo.com (S.S.); ranavirologist@gmail.com (M.S.R.); salmaansharif@hotmail.com (S.S.); meharangez@yahoo.com (M.A.); nisar.nadia@googlemail.com (N.N.); uzmaaamir73@yahoo.com (U.B.A.)

**Keywords:** gastroenteritis, rotavirus, astrovirus, norovirus, parechovirus, Pakistan, molecular epidemiology

## Abstract

Despite substantial interventions in the understanding and case management of acute gastroenteritis, diarrheal diseases are still responsible for a notable amount of childhood deaths. Although the rotavirus is known to cause a considerable burden of pediatric diarrheal cases, the roles of other viruses remain undefined for the Pakistani population. This study was based on tertiary care hospital surveillance, from January 2009 to December 2010, including the detection of rotavirus, norovirus, astrovirus, and human parechovirus in children under the age of five using serological or molecular assays. Rotavirus, human parechovirus, norovirus, and astrovirus were detected in 66%, 21%, 19.5%, and 8.5% subjects, respectively. Human parechovirus genotypes, determined through analysis of VP1 gene sequences, showed a great diversity among co-circulating strains. Eighty percent of hospitalized children had dual or multiple viral infections, while 98% parechovirus positive cases were co-infected with rotavirus. The remarkable diversity of viruses associated with the childhood diarrhea in Pakistan calls for large-scale epidemiological surveys, coupled with case control studies, to ascertain their role in clinical manifestations. In addition, these findings also highlight the need for the implementation of up-to-date health interventions, such as the inclusion of a rotavirus vaccine in routine immunization programs for the improvement of quality in child health care.

## 1. Background

Acute viral gastroenteritis is a major public health problem in children below the age of five [1]. Viruses, including the rotavirus (RV), norovirus (NV), and astrovirus (AstV), are established etiological agents associated with acute gastroenteritis [2]. The rotavirus is the leading cause of severe diarrhea worldwide, and accounts for over 600,000 annual deaths among young children [3,4]. The genus *Rotavirus* belongs to *Reoviridae* family and contains 11 segments of double stranded RNA in its genome. The rotavirus has been classified into seven serogroups (A-G) based on antigenicity of the VP6 protein, as well as patterns of the electrophoretic mobility of the 11 RNA segments. The genetic sequences encoding the outer proteins VP7 and VP4 are used to genotype rotavirus strains into a binary system of classification which serves as a key element of epidemiological and disease burden surveys [5]. To date, 27 G- and 35 P-genotypes have been reported, based on nucleotide variations among VP7 and VP4 gene segments, respectively [6], while the majority of rotavirus infections are caused by G1P [7], G2P [4], G3P [7], G4P [7], and G9P [7] worldwide [7,8].

Human astroviruses usually infect children below the age of three with a prevalence rate ranging 10%–30%; out of these, approximately 33%–65% cases are co-infected with rotavirus and/or norovirus [9,10]. Astrovirus belongs to the family *Astrovirinae*, with two defined genera, *Mamastrovirus* and *Avastrovirus*, infecting mammals and avian species, respectively [11]. The enveloped AstV virion contains positive sense, single stranded RNA that encodes three open reading frames (ORFs); ORF1a, ORF1b, and ORF2 [11,12,13]. In humans, eight well-characterized AstV serotypes (HAstV 1–8) have been reported, in addition to the newly identified species, AstV MLB-1 and 2, AstV-VA1, AstV-HMO-A, and AstV-HMO-B [14]. 

Norovirus is believed to be the source of nearly half of all gastroenteritis outbreaks world-wide and 75%–90% of non-bacterial gastroenteritis outbreaks [15,16,17], causing approximately 200,000 annual deaths in children of <5 years in developing countries [18]. The norovirus belongs to the family *Caliciviridae* and has been classified into five genogroups (G1-GV) based on the sequence variations in the capsid gene region. The genogroups GI, GII, and GIV contain human strains, where GIII infects cattle, and GV contains murine strains [19]. The mature virion contains a positive-sense single stranded RNA genome of 7.5 kb constituting three open reading frames (ORFs) [20].

Recently, other viruses, such as the parechovirus, bocavirus, and saffold virus, have been described in patients with gastroenteritis, although their specific roles as sole or co-pathogen have not been recognized thus far [21,22]. In Pakistan, very few studies explored the viral etiology for gastroenteritis and a majority focused only on the rotavirus [23,24,25,26]. Similarly, there is only a single reported study, based on norovirus prevalence in children admitted to Civil Hospital, Karachi, which was done two decades ago [27]. In addition, only two studies have been conducted on the astrovirus thus far, during the 1990s and in 2013, focusing on gastroenteritis patients from Karachi and Rawalpindi, respectively [27,28]. Our present study presents the first comprehensive analysis of clinical significance and molecular epidemiology of viruses associated with acute dehydrating gastroenteritis in the indigenous Pakistani population. In addition to the more common RV, NV, and AstV, demographic and clinic-epidemiological features of human parechovirus (HPeV) infections are also described. The prevalence and pathogenesis of each HPeV genotype is discussed in relation to their clinical association.

## 2. Methods

### 2.1. Study Population and Sample Collection

This study was conducted at the Department of Virology, National Institute of Health (NIH), Islamabad, which serves as the World Health Organization Collaborating Center for Research and Training in Viral Diagnosis. This work was planned as a cross-sectional study, based on non-probability convenience samples collected from admitted cases who attended the General Hospital in Rawalpindi (a tertiary care hospital) during 2009–2010. The study concept and design were approved by the NIH Internal Review Board. Stool samples were collected after informed written consent from the parents/guardians at the time of sample collection. A pre-tested, duly approved questionnaire form, including the demographic and clinical data, was also completed at the time of sample collection.

From January 2009 to December 2010, 563 stool samples were collected from hospitalized children below five years of age who met the World Health Organizations’ standard case definitions [29], which defines diarrhea as “the passage of three or more loose or liquid stools per day”. Patients who presented with bloody diarrhea during this period were excluded from the study.

### 2.2. Laboratory Investigations for Rotavirus, Astrovirus, Norovirus, and Parechovirus

All samples were analyzed for RV, NV, and AstV, using serological or molecular assays. Rotavirus screenings were performed using ProSpecT™ Rotavirus Microplate Assay (Oxoid Ltd. Wade Road, Basingstoke Hants, UK). Samples were screened for Astrovirus by One-Step Reverse Transcriptase-Polymerase Chain reaction (RT-PCR) assay using primers Mon-269 (5'-CAACTCAGGAAACAGGGTGT-3') and Mon-270 (5'-TCAGATGCATTGTCATTGGT-3'), as described previously [30]. Norovirus detection and genogrouping was performed by real-time PCR assay, as described by Kageyama *et al.* [31]. This assay detected the norovirus by amplifying 85 bp and 98 bp fragment of ORF1-ORF2 junction region using primers COG1F (5'-CGYTGGATGCGNTTYCATGA-3'), COG1R (5'-CTTAGACGCCATCATCATTYAC-3') and COG2F (5'-CARGARBCNATGTTYAGRTGGATGAG-3'), COG2R (5'-TCGACGCCATCTTCATTCACA-3') with probes Ring1 (5'-FAM-AGATYGCGATCYCCTGTCCA-TAMRA-3') and Ring2 (5'-FAM-TGGGAGGGCGATCGCAATCT-TAMRA-3'). 

Parechovirus RNA was directly tested by real time PCR, as described by Nix *et al.* [32]. The 760 bp fragment of the VP1 gene, covering the nucleotide position from 2332 to 3090, according to the prototype strain Harris (GenBank Accession number L02971), was amplified using the primers VP1-parEchoF1 (5'-CCAAAATTCRTGGGGTTC-3') and VP1-parEchoR1 (5'-AAACCYCTRTCTAAATAWGC-3'), as described by Benschop *et al.* [33]. The PCR amplicons of the VP1 gene were purified and sequenced in both directions using a Big Dye Terminator cycle sequencing kit v3.1 (Perkin Elmer-Applied Biosystems, Inc., Foster City, CA, USA).

The sequence data was collected by an ABI Prism Genetic Analyzer (3130xl, Applied Biosystems) and edited using Sequencher v.4.1 (Gene Codes Inc., Ann Arbor, MI, USA). Phylogenetic analyses for parechovirus sequences were performed in comparison to the strains belonging to different genotypes and geographical regions, as retrieved from GenBank. Evolutionary tree and distances (number of base substitutions per site) were generated by the Neighbor Joining method with the Kimura-2 parameter using MEGA 5.0 [34].

Demographic and clinical data were analyzed by SPSS v16.0. Statistical comparisons of qualitative and quantitative clinical parameters were made using Pearson’s Chi-square test and student’s *t*-test, respectively. The comparison of differences between the quantitative clinical parameters, *i.e.*, age, duration of clinical symptoms, number of vomiting and diarrhea episodes per 24 h, duration of clinical symptoms, and duration of treatment, was made using Analysis of Variance (ANOVA) test to infer the association between viral infections (either single or multiple) and their associated clinical conditions. *p-*value ≤ 0.05 was considered statistically significant.

## 3. Results

### 3.1. Prevalence of Specific Viruses

Overall, at least one viral pathogen was detected in 74% (*n* = 417) of the 563 total samples. The highest proportion of enrolled children was positive for rotavirus (66%; *n* = 371) followed by parechovirus (21%; *n* = 118), Norovirus (19.5%; *n* = 110), and astrovirus (8.5%; *n* = 48). There was evidence of dual infection in 48% (*n* = 270) of subjects in varied proportions; (RV + HPeV = 20.4%; RV + NV = 13.1%; RV + AstV = 6.4%; NV + HPeV = 4.8%; AstV + HPeV = 2.1%, and NV + AstV = 1%). Only two cases were infected with all four viruses, whereas triple infection was detected in 6% (*n* = 35) of subjects. The overall mean age of infected children was 11 ± 8.5 months (median = 8) and 60% of infections, irrespective of viral etiology, were found in males. Mean duration of critical symptoms, such as diarrhea and vomiting, was 4.15± 1.7 days. The severity of diarrhea was more pronounced than vomiting, with 7.3 ± 4.2 *vs.* 3.8 ± 3.3 episodes per 24 h, respectively. The average length of hospital treatment was 2.10 ± 1.06 days (range: one to eight days) and all subjects recovered after oral and/or intravenous rehydration therapy.

### 3.2. Clinical Differences between Different Enteric Infections

**Figure 1:**
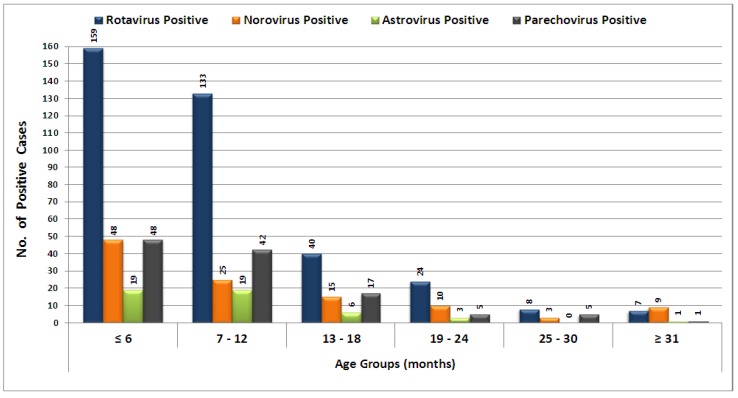
Age-wise distribution of cases that tested positive for rotavirus, parechovirus, norovirus, and astrovirus, during 2009–2010.

Among the clinical manifestations, presence of fever was compared between positive and negative cases and found to be a significant factor in RV, HPeV, and AstV infections (*p* = 0.001, *p* = 0.001 and *p* = 0.013, respectively). Age was also a significant factor for rotavirus infection (*p* = 0.030) when compared among RV positive and negative cases. Among the overall positive subjects (*n* = 417), 42% of infections were found in children below six months of age. Norovirus infections were almost equally distributed among children below and above six months of age (Figure 1).

**Table 1:** Comparison of quantitative clinical parameters between subjects infected with the four different viruses, *i.e.*, rotavirus, parechovirus, norovirus, and astrovirus. Infections were categorized into single and co-infection with dual viruses. *P*-value ≤0.05 was considered significant and are highlighted in yellow. Mean ± SD values are given for each clinical parameter. “S” in superscript indicates significant *p-*values.

Infection Type	Clinical Parameters (Mean ± SD)						
	Age (months)	Duration of Symptoms (days)	Vomiting episodes per 24 hrs	Vomiting duration (days)	Diarrhea episodes per 24 hrs	Diarrhea duration (days)	Duration of Treatment (days)
HPeV + Rotavirus							
HPeV (n = 03)	8 ± 3.7	4.67 ± 1.5	4.67 ± 3	3 ± 2.6	6.69 ± 4.7	4.67 ± 1.5	2.67 ± 2.08
Rotavirus (n = 256)	9.6 ± 7.8	4.21 ± 1.7	3.8 ± 3.4	2.4 ± 2.1	7.8 ± 4.9	4.2 ± 1.9	2.23 ± 1.04
Co-infection (n = 115)	10 ± 7	4.15 ± 1.6	4 ± 2.7	2.7 ± 1.9	7.24 ± 2.8	4.17 ± 1.6	2.12 ± 0.890
*P*-value	**0.003^S^**	0.789	0.684	**0.006^S^**	0.065	0.938	**0.006^S^**
HPeV+Norovirus							
HPeV (n = 91)	9.8 ± 7	4.09 ± 1.6	4.16 ± 2.8	2.63 ± 2.07	7.18 ± 3	4.13 ± 1.7	2.07 ±0.81
Norovirus (n = 83)	12.4 ± 11	4.25 ± 1.9	3.05 ± 3.8	2.5 ± 2.4	6.51 ± 4	4.45 ± 1.9	1.92 ± 1.07
Co-infection (n = 27)	10.37 ± 7.6	4.41 ± 1.5	3.85 ± 2	3 ± 1.6	7.3 ± 3	4.3 ± 1.4	2.32 ± 1.21
*P*-value	0.153	0.786	0.14	0.74	0.333	0.628	0.196
HPeV+Astrovirus							
HPeV (n = 106)	10.3 ± 7.4	4 ± 0.8	4.92 ± 3.6	2.7 ± 1.9	7 ± 2.9	4.18 ± 1.7	2.10 ± 0.99
Astrovirus (n = 36)	11.1 ± 9.4	4.09 ± 1.90	5.45 ± 3.44	3.69 ± 2.1	7.4 ± 4.4	4.7 ± 2.1	2.1 ± 0.99
Co-infection (n = 12)	7.7 ± 6	4.17 ± 1.6	4.7 ± 3	3 ± 2.2	7 ± 2.1	4.7 ± 1.6	2 ± 0.79
*P*-value	0.563	**0.02^S^**	0.062	**0.034^S^**	0.99	0.355	0.967

Comparison of clinical parameters showed that vomiting was a presenting feature in 90% of astrovirus and 80% of parechovirus infections, when compared to rotavirus (74%) and norovirus (72%) positive patients. HPeV co-infections with rotavirus, norovirus, and astrovirus were observed at frequencies of 97.4%, 24.5%, and 25%, respectively (Table 1). To understand the impact of HPeV in multiple viral infections, comparison of clinical parameters between different infection groups was carried out, as presented in Table 1. There was no significant difference of clinical signs among the subjects with HPeV infection compared to those with HPeV plus norovirus infection, for instance, the mean vomiting duration in HPeV positive and HPeV + NV infected children was 2.63 ± 2.07 days and 3 ± 1.6 days (*p* = 0.74), respectively. When comparing between HPeV and HPeV + RV infections, age, vomiting duration, and treatment duration, were found as significant clinical parameters with *p*-values = 0.003, 0.006, and 0.006, respectively. Similarly, HPeV co-infection with astrovirus had a significant effect on the duration of symptoms and vomiting duration (*p* = 0.02 and 0.03, respectively).

### 3.3. Seasonal Trends of Infections with Enteric Viruses

**Figure 2:**
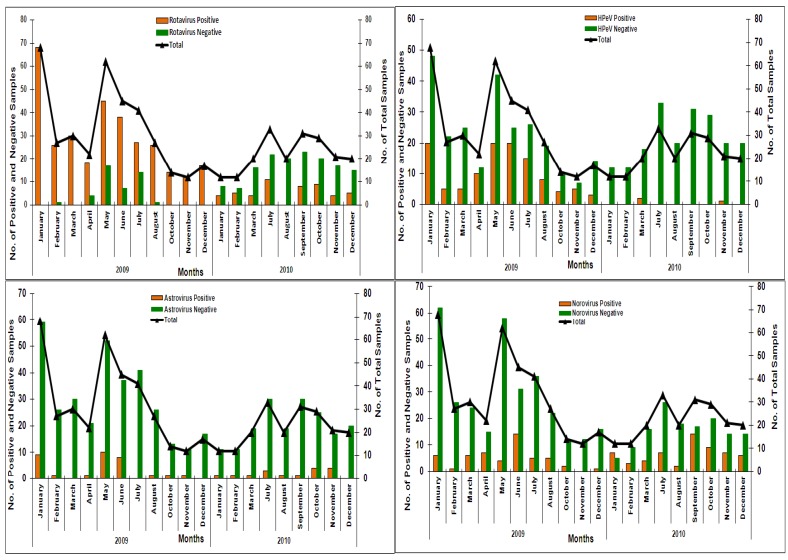
Month-wise distribution of positive, negative, and total cases tested for rotavirus, parechovirus, norovirus, and astrovirus. The months per each year (2009 and 2010) are shown on the X-axis. Number of positive and negative cases are represented on the primary Y-axis and the number of total cases is given on the secondary Y-axis.

No peculiar seasonality trend was noticed among rotavirus, norovirus, astrovirus, and parechovirus infections (Figure 2). In 2009, rotavirus infections were common during the summer months (May–July) but were found throughout the year during 2010. Parechovirus infections were extensively frequent in 2009, with 97% of infections reported between January and December, whereas only three samples (2.5%) were positive in 2010. Norovirus was found almost equally distributed throughout the two-year study period. Similarly, a low level of astrovirus circulation was recorded without any defined seasonal trend.

### 3.4. Phylogenetics and Clinical Significance of HPeV Genotypes

**Figure 3:**
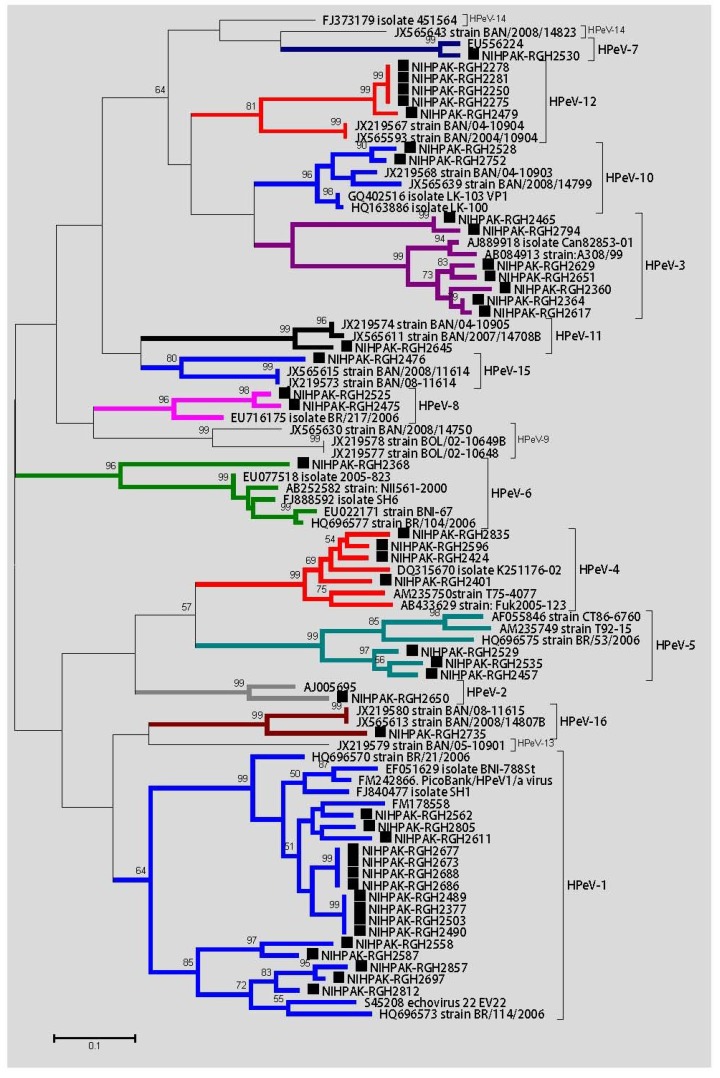
Phylogenetic analysis of human parechovirus strains, detected in hospitalized Pakistani children during January 2009–December 2010. The branch color represents different genotypes, with their names specified after square brackets. HPeV genotypes identified in this study are given NIHPAK-RGH name codes and marked with black squares. The percentage of replicate trees in which the associated taxa clustered together in the bootstrap test (1000 replicates) is shown next to the branches (only bootstrap more than 50 are shown). Scale bar indicates nucleotide substitutions per site.

The parechovirus strains found in our study subjects were further tested to determine their genetic diversity and relatedness to the globally reported strains. Forty-five samples were randomly selected to obtain their partial VP1 gene sequences. Out of the total 16 known genotypes identified thus far, we found 13 in our population with variable proportions (Figure 3); HPeV-1 (35%), HPeV-3 (15.5%), HPeV-4 and -12 (11.1%), HPeV-5 (6.6%), HPeV-8 and 10 (4.4%), and HPeV-2, -6, -7, -11, -15, and -16 (2.2%). Infection with HPeV, other than genotype-1, was found exclusively in the summer season during May-July, however, HPeV-1 infections were distributed around the year. No significant relation of age-to-HPeV-genotype-specific infections was observed. Eighty percent of infections were found in hospitalized children younger than 18 months of age, while none of the subjects over 24 months were found to be positive except two children which were infected with genotypes 3 and 12 of HPeV. 

## 4. Discussion

Every year, there are more than one billion diarrheal episodes with 2.5 million deaths, mainly in underdeveloped regions of the world, experienced by children under five years of age [3,35], in contrast to developed countries, where less than 1% of childhood deaths are associated with diarrhea [3]. Globally, diarrheal infections kill one out of ten children, accounting for 800,000 mortalities, mostly in Africa and South Asia [36]. You *et al.* have described that 32% of the annual 7.6 million deaths in children under the age of five are more concentrated in South Asia [37]. According to a global health survey in 2008, Pakistan is one of the Asian countries that bears the highest number of deaths in children under five [37]. Remarkably, the median incidence of diarrheal disease in children under five in developing countries has not changed since the early 1990s [3]. Therefore, this study was aimed to highlight the role of viral etiologies in gastroenteritis cases in Pakistan; a country with the highest rates of under-five mortality among the 18 countries of the Eastern Mediterranean region, accounting for 464,886 annual deaths [38].

The findings of this study demonstrate that childhood diarrhea in Pakistan is associated with multiple viral infections, with the highest contributions (66%) by the rotavirus followed by parechovirus (21%), norovirus (19.5%), and astrovirus (8.5%). In previous studies, based on analysis of stool samples collected during 1990–1994, the astrovirus and norovirus prevalence in Pakistan was reported at 11% and 10%, respectively [27]. Our findings show a slightly higher infection rate for AstV and NV, supporting their erratic temporal and spatial epidemiology [39]. The figures regarding rotavirus burden are quite significant, for instance, in 2008, the rotavirus was responsible for 453,000 deaths, with over half of these concentrated in five countries, Pakistan, India, Nigeria, Congo, and Ethiopia [40]. Kawai *et al.* (2012) determined that the mortality rate of children under the age of five, attributable to diarrhea, is fairly high in Pakistan with ~74,000 deaths, of which ~23,000 were due to RV infections [41]. As a rotavirus vaccine has not yet been incorporated into the national Expanded Program for Immunization (EPI), it is still a major contributor to the enteric disease burden in our pediatric population. Reports from other developing countries in Asia show similar trends, e.g., Taniuchi *et al.* (2013) found the rotavirus as a substantial contributor of gastroenteritis among Bangladeshi infants, while the role of norovirus and astrovirus infections was relatively insignificant [42]. Likewise, in India, rotavirus mortality figures are fairly high when compared to infections by other viruses, with an estimated 122,000–153,000 RV deaths per annum [43]. Liu *et al.* found that the rotavirus is still the leading cause of childhood hospitalizations due to gastroenteritis in China, and warrants immediate introduction of a rotavirus vaccine to the Chinese pediatric immunization program [44]. In contrast, a significant reduction in diarrhea related national hospital admissions has been recorded after the introduction of a rotavirus vaccine in high-income settings [45,46,47]. Due to scarcity of regional studies focusing on etiological and epidemiological features of vaccine-preventable diseases, we aimed to enhance our existing knowledge about the role of viruses causing enteric infections in our pediatric population, as recommended by the World Health Organization, and to conduct local surveillance studies prior to the introduction of new vaccines in a community [48]. Noticeably, our findings ratify previous reports [23,41] for an early introduction of a rotavirus vaccine into the routine immunization program of Pakistan; a country where ~27% children succumb to diarrhea within their first year of life [49]. In addition, a rotavirus vaccine has been proven to provide equivalent protection against etiologically single or multiple infections of diarrhea [50], thus, reiterating the need for its early introduction. Such interventions against rotavirus disease will certainly improve childhood survival rates, especially in the high-risk regions with poor hygiene and fragile health systems.

Globally, parechovirus has been detected in the full range of severity, including asymptomatic, mild respiratory, and enteric illnesses, as well as conditions with serious outcomes, such as paralysis and neonatal death [51]. To date, 16 genotypes of HPeV (HPeV 1-16) have been identified, although HPeV-1, 2, and 3 account for the majority of infections worldwide [52]. The available data on HPeV epidemiology has highlighted HPeV-1 to 6 as the most common genotypes [52,53,54]. The remaining genotypes, 7–16, have been rarely found, despite sustained HPeV surveillance programs in many developed countries, such as the United States, Japan, and the Netherlands [55,56]. For instance, HPeV-10 (NIHPAK-RGH2528 and NIHPAK-RGH2752) has been found only in Pakistani and Sri Lankan human subjects [57]. HPeV-15 has been exclusively reported in humans from Pakistan (NIHPAK-RGH2476) and from non-human primates in Bangladesh [58]. Similarly, HPeV-11 and 12 have been found in humans in Pakistan (NIHPAK-RGH2645, NIHPAK-RGH, NIHPAK-RGH2278, NIHPAK-RGH2281, NIHPAK-RGH2250, NIHPAK-RGH2275, NIHPAK-RGH2479), Sri Lanka [59], Bolivia [60], and non-human primates in Bangladesh [58]. This indicates that multiple HPeV genotypes are co-circulating in the Pakistani population. To our knowledge, this is the first study targeting patients with gastroenteritis to report such a vast diversity of parechoviruses. In contrast to previous studies that used *in vitro* growth for detection of parechovirus [61,62,63], the remarkable diversity in our reported data may have resulted due to use of a highly sensitive real-time PCR assay, which is known to be 1000 times more sensitive than *in vitro* culture methods [32]. Similarly, the disparity in circulating HPeV genotypes between developed and developing countries may not have been delineated due to paucity of data regarding their global distribution. In addition, such a considerable genetic diversity in the Pakistani population, with multiple HPeV co-infections, merits in-depth case-controlled studies to explore their pathogenic potential and clinical significance. 

The role of HPeV in numerous clinical manifestations, including meningitis, sepsis, and hepatitis, has been discussed [51,64,65], but no causal relationship has been established except with HPeV type 3 being primarily associated with neonatal sepsis [65,66]. Enteric and respiratory infections due to parechoviruses are consistently reported across Asia, Europe, and the Americas [53,64,67]. For instance, in Sri Lanka and Thailand, HPeV infection was reported in 8.3% and 14.6% children, respectively, who attended hospitals due to gastroenteritis, with the detection of different strains, including HPeV-1, 2, 3, 4, 5, 10, and 11 [59,68]. Pham *et al.* examined 477 stool samples of Japanese children between two months to 15 years of age and found 8.1% HPeV infections, mainly of type 1 and 3 [69]. HPeV genotypes 1, 5, 6, and 8 contributed to gastroenteritis in 16.1% subjects out of a total of 335 enrolled Brazilian children [67]. Interestingly, we hereby report the circulation of HPeV genotypes that are rarely found across the globe, e.g., HPeV-2, which was detected in only four Swedish children with gastroenteritis during 1966–1990 [70]. Similarly, a Dutch study reported 37 HPeV infections during the years 2000–2005, but not a single HPeV-2 strain was recovered [33]. In addition, there are only a few case-control studies that analyzed parallel samples from healthy contacts, e.g., in Germany, 538 samples, including control samples from children without enteritis, were tested for HPeV but no significant correlation with the disease was observed [62]. Similar findings have been reported from China, where no association of HPeV was found on comparing positivity between disease and control groups [53]. Few previous studies have explored the rate of HPeV infections utilizing clinical samples of cases which were negative for common enteric viruses like rotavirus, norovirus, adenovirus, and astrovirus. Key findings include detection of HPeV types 1, 2, 3, and 4 in 14.6% Thai children who were found negative for other enteric viruses [68]. In South Korea, 348 samples of gastroenteritis patients were tested and only 2% turned out to be positive for HPeV genotypes 1 and 4 [71]. Similarly, 8.1% of Japanese children, negative for other viruses, had HPeV-1 and 3 infections [69]. In our study, 97.4% of HPeV positive subjects had co-infection, as previously reported [53,59,72]. Although we did not include a control group in our study, the statistical data, based on clinical features of subjects with HPeV alone and those having co-infection with HPeV and any of the three studied viruses (rotavirus, norovirus, and astrovirus) provide no proof of HPeV association to gastroenteritis or diarrhea. Furthermore, year-round circulation of HPeV, without any peculiar epidemiological trend, coupled with considerable genetic diversity in Pakistani children, questions its underlying role in diarrheal illnesses. We conclude from this study, as supported by other researchers [72,73], that mere detection of HPeV in clinical samples does not confirm or establish its role in gastroenteritis. Understandably, such causal deductions are difficult to ascertain without prior knowledge of history of previous illnesses because HPeV, similar to enteroviruses, may continue to shed among asymptomatic individuals for longer periods of time [74].

## 5. Conclusions

Lack of routine and consistent surveillance data limit the true epidemiological profile of HPeV infections, and demands further studies in order to implicate their precise contribution to the enteric infections in our settings. The laboratory diagnostic capacity at major country hospitals and clinical settings in Pakistan should be improved to routinely screen gastroenteritis samples for viral agents to help reduce hospital stay, and limit use of antimicrobial drugs. There is also a dire need to integrate surveillance programs for infectious diseases and scale-up the universal utilization of existing interventions, including robust diagnosis of viral etiologies of enteric infections and inclusion of vaccines like the rotavirus in the country’s Expanded Program on Immunization. Availability of country-specific disease data, and implementation of pertinent disease control protocols, will eventually support Pakistan’s efforts towards the achievement of the United Nations Millennium Development Goal, targeting a two-thirds reduction in childhood mortality by 2015.
